# Vitamin D-related gene polymorphism predict treatment response to pegylated interferon-based therapy in Thai chronic hepatitis C patients

**DOI:** 10.1186/s12876-017-0613-x

**Published:** 2017-04-17

**Authors:** Kessarin Thanapirom, Sirinporn Suksawatamnuay, Wattana Sukeepaisarnjaroen, Pisit Tangkijvanich, Sombat Treeprasertsuk, Panarat Thaimai, Rujipat Wasitthankasem, Yong Poovorawan, Piyawat Komolmit

**Affiliations:** 1 Divisions of Gastroenterology, Department of Medicine, Faculty of Medicine, Chulalongkorn University, King Chulalongkorn Memorial Hospital, Thai Red Cross Society, No. 1873 Rama IV road, Pathumwan District, Bangkok, 10330 Thailand; 2 Center of Excellence in Liver Diseases, King Chulalongkorn Memorial Hospital, Thai Red Cross Society, No. 1873 Rama IV road, Bangkok, 10330 Thailand; 3 Gastroenterology unit, Department of Medicine, Srinagarind Hospital, Faculty of Medicine, Khon Kaen University, No. 123 Mittraparp Highway, Muang District, Khon Kaen, 40002 Thailand; 4 Department of Biochemistry, Faculty of Medicine, Chulalongkorn University, No. 1873 Rama IV road, Bangkok, 10330 Thailand; 5 Center of Excellence in Clinical Virology Department of Pediatrics, Faculty of Medicine, Chulalongkorn University, No. 1873 Rama IV road, Bangkok, 10330 Thailand

**Keywords:** Chronic hepatitis C virus infection, Vitamin D, Gene polymorphisms, Pegylated interferon

## Abstract

**Background:**

Patients with chronic hepatitis C (HCV) infection have high prevalence of vitamin D deficiency. Genome-wide association study data has showed that several genetic variants within vitamin D cascade affect vitamin D function. This study aimed to determine whether genetic polymorphisms of genes in the vitamin D pathway are associated with treatment responses to pegylated interferon (PEG-IFN)-based therapy in patients with chronic HCV infection.

**Methods:**

The study included 623 Thai patients from 2 university hospitals diagnosed with chronic HCV infection who were treated with a PEG-IFN and ribavirin. Patients were genotyped for functional variants on vitamin D synthetic pathway including *GC* (rs4588, rs7041, rs22020, rs2282679)*, CYP2R1* (rs2060793, rs12794714), *CYP27B1* (rs10877012), and *DHCR7* (rs12785878). Pre-treatment predictors of sustained virologic response (SVR) at 24 weeks following discontinuation of therapy were identified using a logistic regression analysis.

**Results:**

SVR was achieved by 60.5% of patients (52.9% with HCV genotype 1; 66.7% with HCV non-genotype 1). In 44.6% of HCV genotype 1-infected patients, only the variant rs12785878 in the *DHCR7* locus was significantly associated with an SVR. HCV genotype 1 patients who had *DHCR7* rs12785878 GT/TT had a higher rate of SVR than those with the GG allele (59.7% vs. 43.4%, *P* = 0.03), but in HCV non-genotype 1-infected patients, the SVR rate did not differ between the two groups (63.3% and 59.1% for GT/TT and GG allele, *P* = 0.54). By multivariate analysis, liver fibrosis stage 0–1 (OR = 5.00; 95% CI, 2.02–12.37; *P* < 0.001), and *DHCR7* rs12785878 GT/TT allele (OR = 2.69; 95% CI, 1.03–7.05; *P* = 0.04) were independent pre-treatment predictors of SVR following PEG-IFN-based therapy in HCV genotype 1 patients. Baseline HCV RNA < 400,000 IU/ml (OR = 1.96; 95% CI, 1.13–3.39; *P* = 0.02) was the only independent predictor of SVR in HCV non-genotype 1 patients. The polymorphisms of *GC*, *CYP2R1* and *CYP27B1* were not associated with treatment outcome even in genotype 1 or non-genotype 1 HCV infection.

**Conclusion:**

The *DHCR7* polymorphism may be a pre-treatment predictive marker for response to PEG-IFN-based therapy in chronic HCV genotype 1 infection.

**Electronic supplementary material:**

The online version of this article (doi:10.1186/s12876-017-0613-x) contains supplementary material, which is available to authorized users.

## Background

Chronic infection with hepatitis C virus (HCV) is a major cause of cirrhosis and hepatocellular carcinoma, with an estimated 185 million people worldwide with chronic HCV infection reported in 2012 [1]. Chronic HCV infection can be treated with the combination therapy of pegylated interferon (PEG-IFN) and ribavirin, which can achieve a sustained virologic response (SVR) in approximately 42%–80% of patients [2, 3]. Despite the development of novel therapy with direct-acting agents (DAAs), these new regimens are expensive and not widely accessible in low-income developing countries, including Thailand. Several patient pre-treatment factors have been demonstrated to assist clinicians in predicting the chance of obtaining an SVR prior to PEG-IFN-based therapy for individual patients with chronic HCV infection [4–6].

Recent studies have shown that vitamin D can act as an immune modulator and regulate hepatic fibrogenesis, in addition to its role in calcium and bone metabolism [7–9]. Most of the vitamin D in the body is synthesized from 7-dehydroxycholesterol in sun-exposed skin. The *DHCR7* gene encodes a reductase that functions as a switch to maintain the balance between 7-dehydrocholesterol for pre-vitamin D3 or cholesterol production [9]. Vitamin D3 undergoes a first enzymatic modification in the liver by cytochrome P-450 family 2, subfamily R, polypeptide 1 (CYP2R1) and generates 25-hydroxyvitamin D (25(OH)D) which is subsequently bound to GC-globulin in the circulation. The second step is hydroxylation, mediated by CYP27B1 in the cells of the kidney and other cells, including immune cells, to produce the active metabolite, 1, 25-dihydroxyvitamin D (1,25(OH)_2_D) [10–13].

Vitamin D deficiency is common in patients with chronic liver disease [14, 15]. Patients with chronic HCV infection have lower mean serum vitamin D levels when compared with age-matched and sex-matched healthy controls [16]. Up to two-thirds of patients with chronic HCV infection may have vitamin D deficiency and one in six patients have severe deficiency, which is three-times the number in the general population [17, 18]. Low serum vitamin D levels may be related to liver fibrosis and a reduced response to PEG-IFN therapy in patients with HCV genotype 1 [16, 19]. Additionally, vitamin D supplementation has been shown to improve the SVR rate in HCV genotype 1 patients treated with PEG-IFN and ribavirin [17, 20, 21]. Findings from large genome-wide association studies (GWAS) and systematic reviews have shown that single nucleotide polymorphisms (SNPs) involved in the vitamin D synthetic pathway, including *GC*, *CYP2R1, CYP27B1* and *DHCR7* can influence serum 25(OH)D levels [22–24]. Previous studies have shown that functional polymorphisms within *CYP27B1-1260* (rs10877012) are associated with poor response to PEG-IFN therapy in European patients with chronic HCV infection, especially with unfavorable *IL28B* rs1279860 CT/TT genotype [18, 25].

However, there are limited data on the association between common SNPs that control vitamin D metabolism and SVR following PEG-IFN therapy for chronic HCV in Thai patients. Therefore, the purpose of this study was to demonstrate whether genetic variants of *DHCR7*, *CYP2R1*, *GC,* and *CYP27B1* are associated with the response to PEG-IFN-based therapy in Thai patients with chronic HCV infection.

## Methods

### Study population

This study was conducted between June 2012 and December 2013 at Chulalongkorn University Hospital (Bangkok, Thailand) and Srinagarind Hospital (Khon Kaen, Thailand). The study included 623 Thai patients with chronic HCV infection, all of whom had compensated liver disease. All the patients included in the study fulfilled the following inclusion criteria: a positive test for anti-HCV antibody, detectable serum HCV RNA, treatment with standard doses and duration (24–72 weeks) of PEG-IFN including PEG-IFN-alfa 2a or 2b in combination with ribavirin. Patients with concomitant human immunodeficiency virus, hepatitis B virus infection, end-stage renal disease and decompensated cirrhosis, patients who were post-liver transplantation, and patients treated with immunosuppressive drugs were excluded from the study. Patient demographic and clinical characteristics at baseline, during and after treatment with a PEG-IFN-based regimen, including age, sex, body mass index (BMI), alcohol drinking history, serum HCV RNA, and biochemical data were recorded. Staging of liver fibrosis was evaluated by tissue histology or measurement of liver stiffness.

The study was conducted in accordance with the ethical principles of the Declaration of Helsinki under Good Clinical Practice. The study protocol was approved by the local Institutional Review Board (IRB). Patients gave written informed consent to participate in the study, according to the requirements of the local ethics committee.

### HCV RNA measurements and definition of virologic response

HCV RNA was quantified using a real-time reverse-transcription polymerase chain reaction (PCR)-based assay (COBAS TaqMan HCV assay, Roche Diagnostics, Basel, Switzerland). HCV genotyping was performed by using the line probe assay INNO-LiPA HCV (Innogenetics, Gent, Belgium).

Rapid virologic response (RVR), early virologic response (EVR) was defined as undetectable HCV RNA at weeks 4 and 12 during PEG-IFN-α therapy. SVR was defined as undetectable HCV RNA in the plasma 24 weeks after the completion of treatment.

### Characterization of vitamin D pathway gene polymorphisms

Genomic DNA was extracted from buffy coat using standard phenol-chloroform method. The eight studied SNPs were genotyped, including *GC* (rs4588 C > A, rs7041 G > T, rs22020 G > A, rs2282679 A > C)*, CYP2R1* (rs2060793 T > C, rs12794714 C > T), *CYP27B1* (rs10877012 C > A), and *DHCR7* (rs12785878 G > T).

The PCR techniques was used and followed by restriction fragment length polymorphism assays. The PCR-specific primer sets were designed and showed in Additional file 1: Table S1. The studied SNPs were selected on the functional of clinical applications [22–24].

### Statistical analysis

Statistical analysis of the data was performed using SPSS software version 22.0 (IBM, New York city, NY, USA). Categorical variables were presented as percentages, and continuous variables were presented as means ± standard deviations (SDs). The association between polymorphisms of vitamin D genes and treatment response was assessed using a Pearson chi-square test. The potential factors different from a *p*-value < 0.1 in the univariate analysis were included in the multivariate analysis based on a stepwise logistic regression model to identify independent predictors of SVR. The assumption of Hardy-Weinberg equilibrium was assessed for all SNPs using χ^2^ test.

## Results

### Patient characteristics

A total of 623 patients with chronic HCV infection treated with a PEG-IFN-based regimen were enrolled in the study. All the patients were Thai. There were 213 (34.2%) women. The mean age was 50.1 ± 9.5 years. There were 287 (46.1%) patients infected with HCV genotype 1, and 278 (44.6%) patients infected with HCV genotype 3. Overall, SVR was achieved in 377 (60.5%) patients, of which 147 (52.9%), and 230 (66.7%) patients were infected with HCV genotype 1 and non-genotype 1, respectively.

The baseline and demographic patient characteristics in patients with and without SVR at 24 weeks following the completion of treatment are summarized in Table 1. In all patients, the SVR rate was affected by age, HCV genotype, histologic stage of liver fibrosis, pre-treatment aspartate transaminase (AST) and HCV RNA.

**Table 1:** Clinical characteristics of patients with chronic hepatitis C virus infection

Baseline characteristics	Non-SVR (*n* = 246)	SVR (*n* = 377)	*P*-value
Female	85 (34.6%)	128 (34.0%)	0.88
Age (years),	51.9 ± 8.4	50.0 ± 10.1	0.01
Body mass index (kg/m^2^)	24.6 ± 3.4	24.5 ± 3.5	0.71
Alcohol drinking	138 (68.7%)	155 (62.5%)	0.17
Diabetes mellitus	51 (25.5%)	55 (22.5%)	0.47
Genotype			
1	131 (53.3%)	147 (39.0%)	<0.005
2	0	1 (0.3%)	
3	101 (41.1%)	186 (49.3%)	
6	14 (5.7%)	43 (11.4%)	
Pre-treatment HCV-RNA (IU/ml)	6.02 ± 0.60	5.81 ± 0.88	0.003
Pre-treatment ALT level (U/L)	106.2 ± 158.3	102.5 ± 75.8	0.98
Pre-treatment AST level (U/L)	87.3 ± 130.2	73.4 ± 52.1	0.046
Advanced fibrosis (stage 2–4)	76 (51.0%)	73 (36.0%)	0.005
PEG-IFN alfa-2a	109 (55.6%)	145 (58.2%)	0.58

Data represent as n (%), mean ± SD. *AST* aspartate aminotransferase, *ALT* alanine aminotransferase, *HCV RNA* hepatitis C virus RNA, *PEG-IFN* pegylated interferon, *SVR* sustained virologic response, *SD* standard deviation

### Prevalence of the SNPs of genes involves in the vitamin D pathway in Thai patients with chronic HCV infection

The genotypic distribution of *GC* (rs4588 C > A, rs7041 G > T, rs22020 G > A, rs2282679 A > C), *CYP2R1* (rs2060793 T > C, rs12794714 C > T), *CYP27B1* (rs10877012 C > A), and *DHCR7* (rs12785878 G > T) in Thai patients with chronic HCV infection are shown in Table 2. Genotypic frequencies did not differ from what was estimated based on the Hardy–Weinberg equilibrium equation (*P* > 0.05).

**Table 2:** Genotypic frequencies of *GC, CYP2R1*, *CYP27B1* and *DHCR7* in Thai chronic hepatitis C patients

	All patients (*n* = 623)	Genotype 1 (*n* = 278)	Non-genotype 1 (*n* = 345)
*GC* rs4588			
CC	345 (60.1%)	162 (62.5%)	183 (58.1%)
CA	196 (34.1%)	78 (30.1%)	118 (37.5%)
AA	33 (5.7%)	19 (7.3%)	14 (4.4%)
*GC* rs7041			
GG	64 (11.1%)	28 (10.8%)	36 (11.4%)
GT	237 (41.3%)	108 (41.7%)	129 (41.0%)
TT	273 (47.6%)	123 (47.5%)	150 (47.6%)
*GC* rs222020			
GG	118 (20.1%)	59 (22.1%)	59 (18.5%)
GA	293 (50.0%)	138 (51.7%)	155 (48.6%)
AA	175 (29.9%)	70 (26.2%)	105 (32.9%)
*GC* rs2282679			
AA	236 (60.2%)	132 (64.4%)	104 (55.6%)
AC	130 (33.2%)	59 (28.8%)	71 (38.0%)
CC	26 (6.6%)	14 (6.8%)	12 (6.4%)
*CYP2R1* rs2060793			
TT	58 (10.2%)	30 (11.6%)	28 (9%)
TC	242 (42.5%)	106 (41.1%)	136 (43.7%)
CC	269 (47.3%)	122 (47.3%)	147 (47.3%)
*CYP2R1* rs12794714			
CC	253 (44.5%)	123 (47.5%)	130 (41.9%)
CT	239 (42.0%)	106 (40.9%)	133 (42.9%)
TT	77 (13.5%)	30 (11.6%)	47 (15.2%)
*CYP27B1* rs10877012			
CC	135 (23.2%)	57 (21.5%)	78 (24.7%)
CA	294 (50.6%)	139 (52.5%)	155 (49.1%)
AA	152 (26.2%)	69 (26.0%)	83 (26.3%)
*DHCR7* rs12785878			
GG	272 (65.9%)	145 (70.0%)	127 (61.7%)
GT	118 (28.6%)	53 (25.6%)	65 (31.6%)
TT	23 (5.6%)	9 (4.3%)	14 (6.8%)

### Association between GC, CYP2R1, CYP27B1 and DHCR7 polymorphisms and SVR

**Fig. 1:**
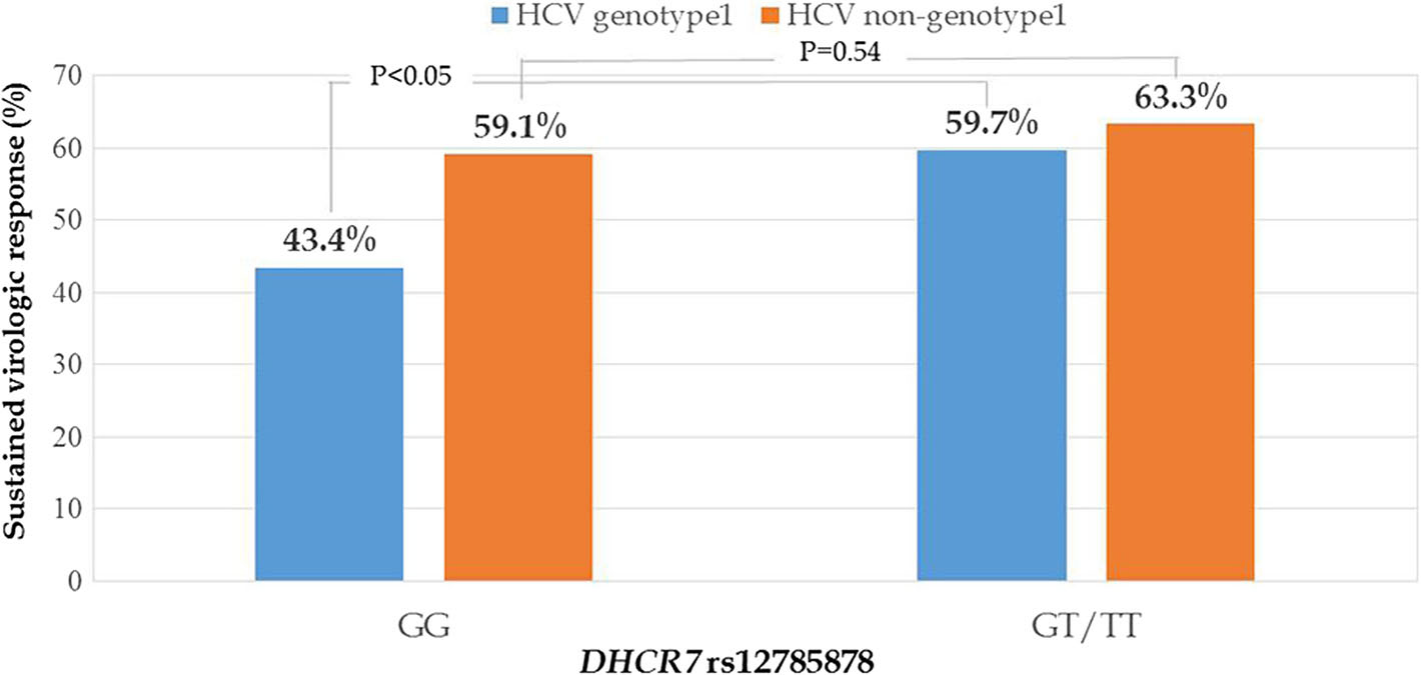
Association of *DHCR7* rs12785878 polymorphism with SVR in Thai patients with chronic HCV infection

The associations between the eight studied SNPs and SVR (classified by HCV genotype) are shown in Additional file 2: Table S2. Among HCV genotype 1-infected patients, *CYP27B1* rs10877012 CA/AA genotype, and *DHCR7* rs12785878 GT/TT genotype was associated with SVR. There was a progressive increase in the rate of SVR according to the *DHCR7* rs12785878 allele (43.4% for GG, 58.5% for GT, and 66.7% for TT), indicating an additive effect of the SNP in HCV genotype 1 infections. Figure 1 shows the rate of SVR according to *DHCR7* rs12785878 polymorphism in patients with chronic HCV infection. There was no association between all studied SNPs and the rate of SVR in patients with HCV non-genotype 1 infection.

### Predictors associated with SVR

Univariate analysis of HCV genotype 1 patients showed that age, liver fibrosis stage 0–1, *CYP27B1* rs10877012 polymorphism, and *DHCR7* rs12785878 polymorphism were related to SVR. Multivariate analysis of HCV genotype 1 patients showed that liver fibrosis stage 0–1 (OR = 5.00; 95% CI, 2.02–12.37; *P* < 0.001), and *DHCR7* rs12785878 GT/TT allele (OR = 2.69; 95% CI, 1.03–7.05; *P* = 0.04) were independent pre-treatment predictors of SVR following PEG-IFN-based therapy (shown in Table 3). In HCV non-genotype 1 patients, pre-treatment AST and HCV RNA were associated with SVR. Logistic regression was used to identify baseline variables for good treatment response and showed baseline HCV RNA < 400,000 IU/ml (OR = 1.96; 95% CI, 1.13–3.39; *P* = 0.02) was the only independent predictor for SVR in these patients.

**Table 3:** Factors associated with sustained virological response to pegylated interferon and ribavirin in chronic hepatitis C virus genotype 1-infected patients

	Univariate analyses		Multivariate analyses	
	Odds ratio (95%CI)	*P*-value	Odds ratio (95%CI)	*P*-value
Age < 50 years	1.93 (1.10–3.38)	0.02	1.76 (0.71–4.37)	0.22
Female	1.26 (0.76–2.09)	0.37		
BMI	1.02 (0.95–1.10)	0.51		
Alcohol drinking	0.82 (0.46–1.46)	0.50		
Diabetes Mellitus	0.78 (0.42–1.45)	0.44		
HCV RNA (log IU/ml)	0.76 (0.55–1.07)	0.11		
ALT	0.99 (0.99–1.00)	0.46		
AST	0.99 (0.99–1.00)	0.44		
Liver fibrosis stage 0–1	3.88 (1.83–8.20)	<0.001	5.00 (2.02–12.37)	<0.001
*CYP27B1* rs10877012 CA/AA	1.67 (0.92–3.02)	0.09	1.24 (0.47–3.29)	0.67
*DHCR7* rs12785878 GT/TT	1.93 (1.05–1.96)	0.032	2.69 (1.03–7.05)	0.04

*AST* aspartate aminotransferase, *ALT* alanine aminotransferase, *BMI* body mass index, *HCV RNA* hepatitis C virus RNA, *95%CI* 95% confidence interval

During treatment, RVR at week 4 and EVR were achieved by 49.1% and 81.7% of HCV genotype 1 patients, respectively, and 62.6% and 77.7% of HCV non-genotype 1 patients, respectively. A higher rate of SVR was obtained among patients who had RVR compared to among those without RVR for both HCV genotype 1 infections (69.8% vs. 30.2%, *P* < 0.001) and non-genotype 1 infections (73.1% vs. 26.9%, *P* < 0.001).

### Performance of DHCR7 rs12785878 polymorphism for predicting SVR in HCV genotype 1 patients

To evaluate the performance of the *DHCR7* polymorphism, liver fibrosis, and RVR in predicting SVR in HCV genotype 1 patients, and to determine the value of the combination of these factors, the sensitivity, specificity, positive predictive value (PPV), and negative predictive value (NPV) were computed (Table 4).

**Table 4:** Performance of *DHCR7* rs12785878 polymorphism, liver fibrosis, and rapid virologic response for predicting sustained virologic response in hepatitis C virus genotype 1-infected patients

	Sensitivity	Specificity	PPV	NPV
*DHCR7* rs12785878 GG/TT	37%	76.6%	59.7%	56.6%
Liver fibrosis stage 0–1	81%	47.6%	66%	66.7%
RVR	64.9%	68.6%	69.8%	63.6%
*DHCR7* rs12785878 GT/TT and RVR	18.1%	95.9%	81%	54.7%
*DHCR7* rs12785878 GT/TT and liver fibrosis stage 0–1	18.4%	97.9%	88.9%	57.2%

*PPV* positive predictive value, *NPV* negative predictive value, *RVR* rapid virologic response

The *DHCR7* rs12785878 GT/TT genotype, liver fibrosis stage 0–1, *DHCR7* GT/TT genotype with RVR, and *DHCR7* GT/TT genotype with fibrosis stage 0–1 were present in 30.0%, 68.3%, 11%, and 9.8% of patients, respectively. The *DHCR7* polymorphism, liver fibrosis, and RVR had similar positive predictive values (PPV). However, the combination of the *DHCR7* polymorphism with RVR or liver fibrosis increased the PPV (81.0–88.9%) and the specificity (95.9–97.9%) for predicting SVR. However, the predictive sensitivity of these factors was low (18.1–18.4%).

## Discussion

The main findings of this study showed that the *DHCR7* rs12785878 GT/TT genotype for vitamin D metabolism was independently associated with a SVR in Thai patients with chronic HCV genotype 1 infection treated with PEG-IFN. Furthermore, the combination of the detection of the *DHCR7* rs1278587*8* variant with the severity of liver fibrosis or RVR increased the predictive value for SVR in HCV genotype 1 patients. These factors may be helpful pre-treatment predictors for optimizing PEG-IFN-based therapy in order to lead to an improved rate of SVR. They may have a particular role in developing countries, including Thailand, where new DAAs therapies are not widely available.

There are increasing evidence on the relevance of vitamin D deficiency and clinical outcomes in patients with chronic HCV infection. Low serum vitamin D levels are associated with liver fibrosis, the presence of extra-hepatic manifestations and poor response to interferon-based therapy in patients with chronic HCV infection. However, the relationship between serum vitamin D level and response to PEG-IFN therapy have been in conflicting from prior studies [16, 26–31]. Functional genetic variations in the vitamin D cascade have been previously shown to impact on clinical outcomes in patients with chronic HCV infection [27, 32]. The expression of the *DHCR7* gene polymorphism has been shown to be associated with the severity of liver fibrosis [27]. Treatment responses following PEG-IFN-based regimens have been previously reported to be associated with common variations in genes in the vitamin D pathway including *CYP27B1*, *VDR*, *CYP2R1*, *GC* and SVR [18, 29, 33–36]. However, to our knowledge, this is the first study that has demonstrated the association between *DHCR7* polymorphisms and treatment responses in patients with chronic HCV infection.

In this study, the *DHCR7* rs12785878 GT/TT genotype was linked to an improved response to PEG-IFN therapy in Thai patients with HCV genotype 1. The *DHCR7* gene encodes 7-dehydrocholesterol reductase, an enzyme involved in the final step of cholesterol synthesis [37]. The SNP, rs12785878, located near the *DHCR7* gene on chromosome 11q12, has been linked to vitamin D serum concentrations in several studies [22, 27]. Findings from large GWAS of a European cohort have shown that the *DHCR7* rs12785878 variant was associated with vitamin D insufficiency [22]. Also, the *DHCR7* GT/TT allele was associated with higher serum vitamin D levels and with decreased liver fibrosis in Caucasian patients with chronic liver disease [38]. In a recent study by Petta and colleagues of patients with chronic HCV genotype 1 infection, the *DHCR7* rs12785878 GT/TT genotype was independently linked to non-severe liver fibrosis [27].

This study has demonstrated that the *DHCR7* GT and TT genotypes are also associated with better treatment responses to PEG-IFN-based regimens in patients with chronic HCV genotype 1-infected patients. The reason for this could be due to their effect on liver fibrosis or due to their enhancement of 7-dehydrocholesterol/pre-vitamin D3 production and immune responses during IFN-based therapy. However, there was no relationship between liver fibrosis and *DHCR7* polymorphisms in the HCV genotype 1 patients in this study. Nevertheless, only half of the patients underwent liver fibrosis staging. It is interesting that why this association is only observed in genotype 1. A possible reason that might explain this finding is patients with chronic HCV non-genotype 1 infection have high SVR rates to PEG-IFN based therapy. The difference among patients with and without the DHCR7 rs12785878 GT/TT are much smaller than genotype 1 patients. There have been no previous studies that have provided data on *DHCR7* rs12785878 genotypic frequencies in Thai populations, but in a 2012 study in Chinese children in northeastern Han Province, frequencies of 25.5%, 46.4%, and 28.1% for GG, GT, and TT genotypes, respectively were reported [39]. The present study showed that functional variants of the genes *GC* (rs4588, rs7041, rs22020, rs2282679), *CYP2R1* (rs2060793, rs12794714) and *CYP27B1* (rs10877012) were not associated with treatment responses in Thai patients with chronic HCV infection.

The findings contrast with the results of previous studies, which showed significant associations between the rate of SVR and a *CYP27B1*-1260 promoter polymorphism (rs10877012) and GC polymorphisms [18, 25, 29]. This finding may be attributable to the differences between studies in patient ethnicity. In addition, the present study analyzed each SNP with SVR, whereas previous studies used genetic models of major allelic combinations. Apart from the *DHCR7* rs12785878 variant, the present study showed that liver fibrosis stage 0–1 and HCV < 400,000 IU/ml at baseline were favorable baseline predictive factors for treatment responses in Thai patients with HCV genotype 1. Several factors, including *IL-28B* genotype, low baseline HCV RNA, less severe liver fibrosis, age and BMI have been reported as pre-treatment predictors of treatment responses among patients undergoing PEG-IFN-based therapy [3, 40, 41]. Despite entering to the new era of chronic HCV therapy with DAAs, which have high rate of SVR, in most developing countries, where DAAs are not affordable by most patients. The PEG-IFN-based regimen remains a good effective option for treatment of chronic HCV infection.

This study had several limitations. Although a large study cohort was evaluated, the study was performed in two main centers in Thailand. Also, the baseline serum 25(OH)D level was not evaluated because it is known to be affected by several variables, including sunlight exposure, season, malabsorption, metabolic rate, dietary intake, and liver function [42]. In addition, the main focus of this study was the effects of genetic variants of genes involved in the vitamin D pathway on treatment outcomes in patients with chronic HCV infection treated with PEG-IFN-based regimens.

## Conclusions

This study has shown that in Thai patients with chronic HCV genotype 1 infection the *DHCR7* rs12785878 GT/TT alleles were independent predictors of improved treatment outcomes with PEG-IFN-based therapy. The *DHCR7* polymorphism may be a predictive marker for good responses to PEG-IFN-based therapy in chronic HCV genotype 1 infection in developing countries, where new DAAs therapies are not widely available.

## Additional files

Additional file 1: Table S1. Restriction Fragment Length Polymorpisms, the primer sequences and polymerase chain reaction conditions, restriction enzymes and product sizes of 8 studied single nucleotide polymorphisms. (DOC 45 kb)

Additional file 2: Table S2. Sustained virologic response in relationship with GC, CYP2R1, CYP27B1 and DHCR7 in chronic hepatitis C patients treated with PEG-IFN-α based regimen. (DOC 59 kb)

## Abbreviations

1,25(OH)2D): 1, 25-dihydroxyvitamin D
25(OH)D: 25-hydroxyvitamin D
95% CI: 95% confidence interval
ALT: Alanine aminotransferase
AST: Aspartate transaminase
BMI: Body mass index
CYP27B1: cytochrome P450 family 27 subfamily B member 1
CYP2R1: Cytochrome P450 family 2 subfamily R member 1
DAA: Direct-acting agents
DHCR7: 7-dehydrocholesterol reductase
DNA: Deoxyribonucleic Acid
EVR: Early virologic response
GC: Vitamin D binding protein
GWAS: Genome-wide association studies
HCV: Hepatitis C virus
IRB: Institutional review board
NPV: Negative predictive value
OR: Odd ratio
PCR: Polymerase chain reaction
PEG-IFN: Pegylated interferon
PPV: Positive predictive value
RNA: Ribonucleic acid
RVR: Rapid virologic response
SNP: Single nucleotide polymorphism
SVR: Sustained virologic response
VDR: Vitamin D receptor

## References

[1] Mohd Hanafiah K, Groeger J, Flaxman AD, Wiersma ST (2013). Global epidemiology of hepatitis C virus infection: new estimates of age-specific antibody to HCV seroprevalence. Hepatology.

[2] Manns MP, McHutchison JG, Gordon SC, Rustgi VK, Shiffman M, Reindollar R, Goodman ZD, Koury K, Ling M, Albrecht JK (2001). Peginterferon alfa-2b plus ribavirin compared with interferon alfa-2b plus ribavirin for initial treatment of chronic hepatitis C: a randomised trial. Lancet.

[3] Fried MW, Shiffman ML, Reddy KR, Smith C, Marinos G, Goncales FL, Haussinger D, Diago M, Carosi G, Dhumeaux D (2002). Peginterferon alfa-2a plus ribavirin for chronic hepatitis C virus infection. N Engl J Med.

[4] Kau A, Vermehren J, Sarrazin C (2008). Treatment predictors of a sustained virologic response in hepatitis B and C. J Hepatol.

[5] Manns MP, Wedemeyer H, Cornberg M (2006). Treating viral hepatitis C: efficacy, side effects, and complications. Gut.

[6] Berg T, Sarrazin C, Herrmann E, Hinrichsen H, Gerlach T, Zachoval R, Wiedenmann B, Hopf U, Zeuzem S (2003). Prediction of treatment outcome in patients with chronic hepatitis C: significance of baseline parameters and viral dynamics during therapy. Hepatology.

[7] White JH (2012). Vitamin D metabolism and signaling in the immune system. Rev Endocr Metab Disord.

[8] von Essen MR, Kongsbak M, Schjerling P, Olgaard K, Odum N, Geisler C (2010). Vitamin D controls T cell antigen receptor signaling and activation of human T cells. Nat Immunol.

[9] Holick MF (2007). Vitamin D deficiency. N Engl J Med.

[10] Cheng JB, Levine MA, Bell NH, Mangelsdorf DJ, Russell DW (2004). Genetic evidence that the human CYP2R1 enzyme is a key vitamin D 25-hydroxylase. Proc Natl Acad Sci U S A.

[11] Rahman AH, Branch AD (2013). Vitamin D for your patients with chronic hepatitis C?. J Hepatol.

[12] Takeyama K, Kitanaka S, Sato T, Kobori M, Yanagisawa J, Kato S (1997). 25-Hydroxyvitamin D3 1alpha-hydroxylase and vitamin D synthesis. Science.

[13] Haussler MR, Whitfield GK, Haussler CA, Hsieh JC, Thompson PD, Selznick SH, Dominguez CE, Jurutka PW (1998). The nuclear vitamin D receptor: biological and molecular regulatory properties revealed. J Bone Miner Res.

[14] Arteh J, Narra S, Nair S (2010). Prevalence of vitamin D deficiency in chronic liver disease. Dig Dis Sci.

[15] Fisher L, Fisher A (2007). Vitamin D and parathyroid hormone in outpatients with noncholestatic chronic liver disease. Clin Gastroenterol Hepatol.

[16] Petta S, Camma C, Scazzone C, Tripodo C, Di Marco V, Bono A, Cabibi D, Licata G, Porcasi R, Marchesini G (2010). Low vitamin D serum level is related to severe fibrosis and low responsiveness to interferon-based therapy in genotype 1 chronic hepatitis C. Hepatology.

[17] Bitetto D, Fattovich G, Fabris C, Ceriani E, Falleti E, Fornasiere E, Pasino M, Ieluzzi D, Cussigh A, Cmet S (2011). Complementary role of vitamin D deficiency and the interleukin-28B rs12979860 C/T polymorphism in predicting antiviral response in chronic hepatitis C. Hepatology.

[18] Lange CM, Bojunga J, Ramos-Lopez E, von Wagner M, Hassler A, Vermehren J, Herrmann E, Badenhoop K, Zeuzem S, Sarrazin C (2011). Vitamin D deficiency and a CYP27B1-1260 promoter polymorphism are associated with chronic hepatitis C and poor response to interferon-alfa based therapy. J Hepatol.

[19] Kitson MT, Dore GJ, George J, Button P, McCaughan GW, Crawford DH, Sievert W, Weltman MD, Cheng WS, Roberts SK (2013). Vitamin D status does not predict sustained virologic response or fibrosis stage in chronic hepatitis C genotype 1 infection. J Hepatol.

[20] Yokoyama S, Takahashi S, Kawakami Y, Hayes CN, Kohno H, Tsuji K, Aisaka Y, Kira S, Yamashina K, Nonaka M (2014). Effect of vitamin D supplementation on pegylated interferon/ribavirin therapy for chronic hepatitis C genotype 1b: a randomized controlled trial. J Viral Hepat.

[21] Abu-Mouch S, Fireman Z, Jarchovsky J, Zeina AR, Assy N (2011). Vitamin D supplementation improves sustained virologic response in chronic hepatitis C (genotype 1)-naive patients. World J Gastroenterol.

[22] Wang TJ, Zhang F, Richards JB, Kestenbaum B, van Meurs JB, Berry D, Kiel DP, Streeten EA, Ohlsson C, Koller DL (2010). Common genetic determinants of vitamin D insufficiency: a genome-wide association study. Lancet.

[23] Ahn J, Yu K, Stolzenberg-Solomon R, Simon KC, McCullough ML, Gallicchio L, Jacobs EJ, Ascherio A, Helzlsouer K, Jacobs KB (2010). Genome-wide association study of circulating vitamin D levels. Hum Mol Genet.

[24] McGrath JJ, Saha S, Burne TH, Eyles DW (2010). A systematic review of the association between common single nucleotide polymorphisms and 25-hydroxyvitamin D concentrations. J Steroid Biochem Mol Biol.

[25] Lange CM, Bibert S, Kutalik Z, Burgisser P, Cerny A, Dufour JF, Geier A, Gerlach TJ, Heim MH, Malinverni R (2012). A genetic validation study reveals a role of vitamin D metabolism in the response to interferon-alfa-based therapy of chronic hepatitis C. PLoS One.

[26] Terrier B, Jehan F, Munteanu M, Geri G, Saadoun D, Sene D, Poynard T, Souberbielle JC, Cacoub P (2012). Low 25-hydroxyvitamin D serum levels correlate with the presence of extra-hepatic manifestations in chronic hepatitis C virus infection. Rheumatology (Oxford).

[27] Petta S, Grimaudo S, Marco VD, Scazzone C, Macaluso FS, Camma C, Cabibi D, Pipitone R, Craxi A (2013). Association of vitamin D serum levels and its common genetic determinants, with severity of liver fibrosis in genotype 1 chronic hepatitis C patients. J Viral Hepat.

[28] White DL, Tavakoli-Tabasi S, Kanwal F, Ramsey DJ, Hashmi A, Kuzniarek J, Patel P, Francis J, El-Serag HB (2013). The association between serological and dietary vitamin D levels and hepatitis C-related liver disease risk differs in African American and white males. Aliment Pharmacol Ther.

[29] Falleti E, Bitetto D, Fabris C, Fattovich G, Cussigh A, Cmet S, Ceriani E, Fornasiere E, Pasino M, Ieluzzi D (2012). Vitamin D binding protein gene polymorphisms and baseline vitamin D levels as predictors of antiviral response in chronic hepatitis C. Hepatology.

[30] Loftfield E, O’Brien TR, Pfeiffer RM, Howell CD, Horst R, Prokunina-Olsson L, Weinstein SJ, Albanes D, Morgan TR, Freedman ND (2016). Vitamin D status and virologic response to HCV therapy in the HALT-C and VIRAHEP-C Trials. PLoS One.

[31] Garcia-Alvarez M, Pineda-Tenor D, Jimenez-Sousa MA, Fernandez-Rodriguez A, Guzman-Fulgencio M, Resino S (2014). Relationship of vitamin D status with advanced liver fibrosis and response to hepatitis C virus therapy: a meta-analysis. Hepatology.

[32] Wu MP, Zhang JW, Huang P, Han YP, Zhang Y, Peng ZH, Wang J, Zhu P, Su J, Yu RB (2016). Genetic variations in vitamin D receptor were associated with the outcomes of hepatitis C virus infection among Chinese population. J Hum Genet.

[33] Lange CM, Bojunga J, Ramos-Lopez E, von Wagner M, Hassler A, Vermehren J, Herrmann E, Badenhoop K, Zeuzem S, Sarrazin C (2010). Vitamin D deficiency and a CYP27B1-1260 promoter polymorphism are associated with chronic hepatitis C and poor response to interferon-alfa based therapy. J Hepatol.

[34] El-Derany MO, Hamdy NM, Al-Ansari NL, El-Mesallamy HO (2016). Integrative role of vitamin D related and Interleukin-28B genes polymorphism in predicting treatment outcomes of Chronic Hepatitis C. BMC Gastroenterol.

[35] Garcia-Martin E, Agundez JA, Maestro ML, Suarez A, Vidaurreta M, Martinez C, Fernandez-Perez C, Ortega L, Ladero JM (2013). Influence of vitamin D-related gene polymorphisms (CYP27B and VDR) on the response to interferon/ribavirin therapy in chronic hepatitis C. PLoS One.

[36] Hung CH, Hu TH, Lu SN, Chen CH, Wang JH, Lee CM (2016). Association of vitamin D receptor gene polymorphisms with response to peginterferon plus ribavirin in Asian patients with chronic hepatitis C. J Formos Med Assoc.

[37] Prabhu AV, Luu W, Li D, Sharpe LJ, Brown AJ (2016). DHCR7: A vital enzyme switch between cholesterol and vitamin D production. Prog Lipid Res.

[38] Grunhage F, Hochrath K, Krawczyk M, Hoblinger A, Obermayer-Pietsch B, Geisel J, Trauner M, Sauerbruch T, Lammert F (2012). Common genetic variation in vitamin D metabolism is associated with liver stiffness. Hepatology.

[39] Zhang Y, Wang X, Liu Y, Qu H, Qu S, Wang W, Ren L (2012). The GC, CYP2R1 and DHCR7 genes are associated with vitamin D levels in northeastern Han Chinese children. Swiss Med Wkly.

[40] Conjeevaram HS, Fried MW, Jeffers LJ, Terrault NA, Wiley-Lucas TE, Afdhal N, Brown RS, Belle SH, Hoofnagle JH, Kleiner DE (2006). Peginterferon and ribavirin treatment in African American and Caucasian American patients with hepatitis C genotype 1. Gastroenterology.

[41] Tanaka Y, Nishida N, Sugiyama M, Kurosaki M, Matsuura K, Sakamoto N, Nakagawa M, Korenaga M, Hino K, Hige S (2009). Genome-wide association of IL28B with response to pegylated interferon-alpha and ribavirin therapy for chronic hepatitis C. Nat Genet.

[42] Rosen CJ (2011). Clinical practice. Vitamin D insufficiency. N Engl J Med.

