# Supplementary material for: Vitamin D-related gene polymorphism predict treatment response to pegylated interferon-based therapy in Thai chronic hepatitis C patients
Source: BMC Gastroenterol. 2017 Apr 17;17:54. doi: 10.1186/s12876-017-0613-x (PMC5392932; doi:10.1186/s12876-017-0613-x)
Supplement: Supplementary file 1 — Restriction Fragment Length Polymorpisms, the primer sequences and polymerase chain reaction conditions, restriction enzymes and product sizes of 8 studied single nucleotide polymorphisms. (DOC 45 kb) [file 12876_2017_613_MOESM1_ESM.doc]

**Table 1s**: Restriction Fragment Length Polymorpisms, the primer sequences and polymerase chain reaction conditions, restriction enzymes and product sizes of 8 studied single nucleotide polymorphisms.

| **Gene** | **SNP ID** | **primer sequence** | **Temp. annealing (°C)** | **restriction enzyme** | **allele** | **product size (bp)** |
| --- | --- | --- | --- | --- | --- | --- |
| *CYP27B1* | rs10877012 | F: 5' TGA CCT TCA ATT CCA GAA CTT CA 3' | 58 | *Hinf*I | C | 151, 104 |
|  |  | R: 5' GGT GGC GTA TGC CTG TAG TG 3' |  |  | A | 255 |
| *CYP2R1* | rs2060793 | F: 5' CCT TGA TAT TTC CTC TGT TTG 3' | 58 | *Hinf*I | T | 285 |
|  |  | R: 5' TCG CTG TCT CTC TGA TTA TCT 3' |  |  | C | 194, 91 |
|  | rs12794714 | F: 5' CGC TCT TCC TGC TGC TCT TC  3' | 58 | *Fok*I | C | 240, 110 |
|  |  | R: 5' GCG TCG AGG ACT TCT CCC TTC 3' |  |  | T | 353 |
| *GC* | rs7041 | F: 5' TAC CAC AGG TAT AGA ATT TT 3' | 53 | *Hae*III | G | 183, 121 |
|  |  | R: 5' AGT GGA GGG TTA CAT TTT CCT 3' |  |  | T | 340 |
|  | rs4588 | F: 5' TAC CAC AGG TAT AGA ATT TT 3' | 53 | *Btg*I | C | 190, 114 |
|  |  | R: 5' AGT GGA GGG TTA CAT TTT CCT 3' |  |  | A | 340 |
|  | rs222020 | F: 5' TTT GAC GTT AGG GCT TCA TCA ACT CAA TGG GCA AAA AAT TCA ATC G 3' | 57 | *Taq*I | G | 330 |
|  |  | R: 5' AGC GAG CTG GCT AGG AGT TGT C 3' |  |  | A | 286, 44 |
|  | rs2282679 | F: 5' TGG CAC TGA CTC TGG CTC TGA 3' | 58 | *Fok*I | T | 205, 61 |
|  |  | R: 5' CTC CCT TCA TGG AAA CAC CTT G 3' |  |  | C | 266 |
| *DHCR7* | rs12785878 | F: 5' CTG TCT TCT CTT AGG AGG TT 3' | 56 | *Taq*I | G | 250, 124 |
|  |  | R: 5' CAA GCA GCA GAC AGG ACA TGA 3' |  |  | T | 374 |
