# Supplementary material for: Vitamin D-related gene polymorphism predict treatment response to pegylated interferon-based therapy in Thai chronic hepatitis C patients
Source: BMC Gastroenterol. 2017 Apr 17;17:54. doi: 10.1186/s12876-017-0613-x (PMC5392932; doi:10.1186/s12876-017-0613-x)
Supplement: Supplementary file 2 — Sustained virologic response in relationship with GC, CYP2R1, CYP27B1 and DHCR7 in chronic hepatitis C patients treated with PEG-IFN-α based regimen. (DOC 59 kb) [file 12876_2017_613_MOESM2_ESM.doc]

**Table 2s: Sustained virologic response in relationship with *GC,* *CYP2R1*, *CYP27B1* and *DHCR7* in chronic hepatitis C patients treated with PEG-IFN-α based regimen.**

|  | **HCV genotype 1** | | | | **HCV non-genotype 1** | | | |
| --- | --- | --- | --- | --- | --- | --- | --- | --- |
|  | **Non-SVR (n=131)** | **SVR (n=147)** | **Odds ratio (95% CI)** | **p-value** | **Non-SVR (n=115)** | **SVR (n=230)** | **Odds ratio (95% CI)** | **p-value** |
| ***GC*** **rs4588** |  |  |  |  |  |  |  |  |
| CC | 82 (66.1%) | 80 (59.3%) | 0.85 (0.4-2.0) | 0.71 | 54 (54.0%) | 129 (60.0%) | 1.28 (0.8-2.1) | 0.32 |
| CA | 31 (25.0%) | 47 (34.8%) |  |  | 41 (41.0%) | 77 (35.8%) |  |  |
| AA | 11 (8.9%) | 8 (5.9%) |  |  | 5 (5.0%) | 9 (4.2%) |  |  |
| ***GC* rs7041** |  |  |  |  |  |  |  |  |
| GG | 12 (9.7%) | 16 (11.9%) | 1.07 (0.5-2.4) | 0.87 | 10 (10%) | 26 (12.1%) | 1.24 (0.6-2.7) | 0.59 |
| GT | 54 (43.5%) | 54 (40.0%) |  |  | 42 (42.0%) | 87 (40.5%) |  |  |
| TT | 58 (46.8%) | 65 (48.1%) |  |  | 48 (48.0%) | 102 (47.4%) |  |  |
| ***GC*** **rs222020** |  |  |  |  |  |  |  |  |
| GG | 31 (24.4%) | 28 (20.0%) | 0.71 (0.3-1.7) | 0.44 | 19 (18.1%) | 40 (18.7%) | 1.04 (0.6-1.9) | 0.90 |
| GA | 66 (52.0%) | 72 (51.4%) |  |  | 56 (53.3%) | 99 (46.3%) |  |  |
| AA | 30 (23.6%) | 40 (28.6%) |  |  | 30 (28.6%) | 75 (35.0%) |  |  |
| ***GC*** **rs2282679** |  |  |  |  |  |  |  |  |
| AA | 61 (68.5%) | 71 (61.2%) | 0.63 (0.3-1.5) | 0.30 | 39 (53.4%) | 65 (57.0%) | 1.16 (0.6-2.1) | 0.63 |
| AC | 20 (22.5%) | 39 (33.6%) |  |  | 28 (38.4%) | 43 (37.7%) |  |  |
| CC | 8 (9.0% | 6 (5.2%) |  |  | 6 (8.2%) | 6 (5.3%) |  |  |
| ***CYP2R1* rs2060793** |  |  |  |  |  |  |  |  |
| TT | 15 (12.2%) | 15 (11.1%) | 0.90 (0.4-1.9) | 0.79 | 7 (7.1%) | 21 (9.9%) | 1.44 (0.6-3.5) | 0.42 |
| TC | 54 (43.9%) | 52 (38.5%) |  |  | 45 (45.5%) | 91 (42.9%) |  |  |
| CC | 54 (43.9%) | 68 (50.4%) |  |  | 47 (47.5%) | 100 (47.2%) |  |  |
| ***CYP2R1* rs12794714** |  |  |  |  |  |  |  |  |
| CC | 53 (42.7%) | 70 (51.9%) | 1.44 (0.9-2.3) | 0.14 | 47 (47.0%) | 83 (39.5%) | 0.74 (0.4-1.2) | 0.21 |
| CT | 57 (46.0%) | 49 (36.3%) |  |  | 44 (44.0%) | 89 (42.4%) |  |  |
| TT | 14 (11.3%) | 16 (11.9%) |  |  | 9 (9.0%) | 38 (18.1%) |  |  |
| ***CYP27B1* rs10877012** |  |  |  |  |  |  |  |  |
| CC | 33 (26.0%) | 24 (17.4%) | 0.60 (0.3-1.1) | 0.09 | 30 (29.4%) | 48 (22.4%) | 0.69 (0.4-1.2) | 0.18 |
| CA | 59 (46.5%) | 80 (58.0%) |  |  | 47 (46.1%) | 108 (50.5%) |  |  |
| AA | 35 (27.6%) | 34 (24.6%) |  |  | 25 (24.5%) | 58 (27.1%) |  |  |
| ***DHCR7* rs12785878** |  |  |  |  |  |  |  |  |
| GG | 82 (76.6%) | 63 (63.0%) | 0.52 (0.3-0.9) | 0.03 | 52 (64.2%) | 75 (60.0%) | 1.19 (0.7-2.1) | 0.55 |
| GT | 22 (20.6%) | 31 (31.0%) |  |  | 24 (29.6%) | 41 (32.8%) |  |  |
| TT | 3 (2.8%) | 6 (6.0%) |  |  | 5 (6.2%) | 9 (7.2%) |  |  |
